# Impact of the COVID-19 pandemic on burnout and perceived workplace quality among addiction treatment providers

**DOI:** 10.1186/s13722-023-00361-6

**Published:** 2023-01-20

**Authors:** Andrea Fentem, Raven Riordan, Christine Doroshenko, Xiao Li, Erin Kasson, Devin Banks, Rachel P. Winograd, Patricia Cavazos-Rehg

**Affiliations:** 1 Department of Psychiatry, Washington University School of Medicine, 660 South Euclid Avenue, Box 8134, St. Louis, MO 63110 USA; 2 Department of Psychological Sciences, University of Missouri – St. Louis, One University Blvd. 325 Stadler Hall, St. Louis, MO 63121 USA; 3 Missouri Institute of Mental Health, University of Missouri St. Louis, 43 Benton Court, St. Louis, MO 63121 USA

**Keywords:** COVID-19, Burnout, Stress, Substance use, Providers

## Abstract

**Background:**

This study examines the impact of the COVID-19 pandemic on work satisfaction, work-related stress, and perceived work quality among substance use treatment providers to better understand challenges faced among this group during the pandemic.

**Methods:**

Participants of this study were 91 addiction treatment providers (e.g., therapists, physicians, community support specialists, administrative staff) recruited from various treatment facilities (e.g., inpatient and outpatient settings). Mixed method analyses were conducted to assess self-reported burnout, sources of work-related stress, and perceived work quality during the pandemic. Responses from providers reporting COVID-19 related decreases in work quality were compared to responses from providers who reported their quality of work had increased or remained the same.

**Results:**

Results demonstrated half of providers (51%) reported their quality of work had decreased. This perceived decrease in quality of work was associated with higher levels of emotional exhaustion (*M* = 17.41 vs. *M* = 12.48, *p* = 0.002), workplace stress (*M* = 42.80 vs. *M* = 30.84, *p* = 0.001), as well as decreased enjoyment of work (83% vs. 51%, *p* = 0.001) and decreased personal accomplishment (*M* = 20.64 vs. *M* = 23.05 *p* = 0.001). Qualitative investigations further illustrated that increased hours, changes in work schedules, work-life balance challenges, difficulties with client communication, and increased client needs were contributing factors increasing stress/burnout and decreasing perceived work quality.

**Conclusions:**

Addiction treatment providers experience high levels of burnout and workplace stress. Additionally, many individuals perceived a decrease in their quality of work during the COVID-19 pandemic. Addiction treatment facility administration should address these challenges to support the well-being of clinical staff and the clients they serve both during and after the COVID-19 pandemic.

**Supplementary Information:**

The online version contains supplementary material available at 10.1186/s13722-023-00361-6.

## Introduction

Addiction treatment providers offer counseling, medical care, and community supports for those struggling with substance use disorders (SUDs) and often are tasked with providing support for associated behavioral health conditions (e.g., depression, anxiety) and environmental suffering (e.g., loss of housing, transportation issues). Helping clients achieve stability in the face of high risk of relapse [14, 29, 34] and comorbid mental health concerns [2] may make this job emotionally burdensome for the clinician [4]. Additionally, addiction treatment providers tend to work under difficult conditions, which include low salaries, high staff turnover, demanding workloads, and limited opportunities for career advancement—all of which contribute to burnout [33, 42]. The financial, psychological, and physical impact of the current COVID-19 pandemic on providers, treatment systems, and the people they serve have only exacerbated these challenges [9, 43].

Burnout among addiction treatment providers is concerning because of its association with job turnover and increased instability within addiction treatment agencies [38], which in turn have negative impacts on continuity and standard of care for clients [19]. Burnout is a psychological condition involving a prolonged response to enduring interpersonal stressors [24]. It affects individuals by resulting in various forms of job withdrawal, such as decreased work quality, lack of connection with clients, and low job satisfaction [24, 39]. Burnout has been reported as a prevalent problem among addiction treatment providers and other providers of mental health and substance use care [12, 24]. For example, a meta-analysis found that studies reported 21–67% of mental health and substance use providers report high levels of burnout [31], and consequences of such burnout include poor provider physical and mental health, low workplace quality, decreased quality of care, and poor client recovery [15, 23].

Those who treat substance misuse have been under tremendous stress during the COVID-19 pandemic, and the rapid adaptations to the pandemic needed to ensure continued access to treatment have exacerbated this stress [8]. For instance, many treatment providers quickly transitioned services to telehealth platforms, but these transitions came with significant difficulties such as trouble establishing and/or maintaining patient-provider relationships, the inability to perform adequate physical exams, a lack of technology necessary for telehealth visits, and limited experience with telehealth technology [7, 8]. In addition, many clinicians experience the same anxieties that impact much of society: worry surrounding their own risk of getting sick or bringing the virus home to their families, stress from limited access to childcare or home schooling responsibilities, concern for elderly family members, and strain from social isolation [40]. Indeed, over half (51%) of healthcare professional respondents (i.e., mental health providers, nurses, addiction treatment providers, etc.) across 33 countries reported emotional exhaustion related to their work during the COVID-19 pandemic, with the U.S. reporting the highest burnout at a rate of 63% [30].

Given increases in burnout and challenges to addiction treatment provision during the COVID-19 pandemic, the objective of this study was to understand the impact of the pandemic on addiction treatment providers. Although there are several studies that predict burnout among addiction treatment providers [4, 5, 28, 45], no study has yet examined how the COVID-19 pandemic has impacted addiction treatment providers’ perceived enjoyment and quality of work and the current factors associated with their experiences of burnout during this time. We hypothesized that addiction treatment providers would report decreased levels of enjoyment and quality of work related to the impact of the COVID-19 pandemic. Findings from this study will inform infrastructure needed to support addiction providers as the long-term impacts of the pandemic continue to affect substance use treatment systems and delivery of care.

## Methods

### Participants

The present study is based on data from participants who were recruited from various facilities (N = 9) within Missouri: outpatient and inpatient programs. The full sample included 91 providers (e.g., LCSW, nurses, support specialists, and administration)) who were providing substance use services during the COVID-19 pandemic. Survey data was collected from April to August 2020 during the height of the COVID-19 lockdown restrictions.

### Procedure

Administration from each participating facility provided study information to all agency staff. Those who were interested in participating contacted the research team and consented via email or phone. Participants completed a survey concerning their views on how the COVID-19 pandemic has impacted their work and personal life. All responses were self-reported using the online Research Electronic Data Capture (REDCap) system, which is a secure, web-based application that can be used either on a computer or mobile device. Research staff reviewed the informed consent document with eligible participants via phone or in-person and participants provided verbal or written consent to join the study. This study was approved by the study team’s Institutional Review Board (IRB # 202006022).

### Measures

#### 2.3.1 Demographics

Among all participants, socio-demographic information was assessed, including age (18–30 vs. 31–50 vs. 51 or above years old), gender (Cisgender Male, Cisgender Female, or Other gender), race/ethnicity (Caucasian vs. African American vs, Others), marital status (married vs. other), education level (lower than bachelor’s degree vs. bachelor’s degree vs, higher than bachelor’s degree), and having any kids (no vs. yes).

#### 2.3.2 The Maslach Burnout Inventory-Human Services Survey (MBI-HSS)

The MBI-HSS has become the gold standard to assess burnout in health-related fields [22, 26]. It evaluates the three aspects of burnout—emotional exhaustion (EE), personal accomplishment (PA), and depersonalization (DP). MBI-HSS consists of 22 questions, of which 9 evaluate EE, 5 evaluate DP, and 8 evaluate PA. The internal consistency score overall among the current sample was 0.836. For three aspects of burnout, the scores were 0.936, 0.712, and 0.797, respectively. Each item is answered on a 7-point Likert scale ranging from “never” (= 0) to “daily” (= 6). The composite scores for the three aspects of burnout were computed by averaging the scores for the corresponding questions. Summary scores were created for each subscale variable with higher scores indicating higher levels of EE, PA, and DP. The potential score range was 0 to 36 for EE, 0 to 32 for PA, and 0 to 20 for DP. The reliability of all items measured by Cronbach’s alpha index is 0.8 [20].

#### 2.3.3 Workplace Stress Survey (WSS)

The American Institute of Stress (AIS) created the WSS to assess employee stress levels [1]. Survey participants were asked to assign a number from 1 to 10 for statements that describe amount of work stress and work satisfaction. Summary scores were created for each subscale variable with higher scores indicating higher levels of workplace stress.

#### 2.3.4 Impact of the COVID-19 pandemic

The study’s research team developed four questions to address how the COVID-19 pandemic has impacted the participant’s perceived quality and enjoyment of work while working at an addiction treatment facility. Participants progressed through two stages of responses. In the first stage, participants were queried to respond to a quantitative item asking, “How has the COVID-19 pandemic impacted the quality of your work as a provider?” with forced choice options of “Quality of my work has increased”, “Quality of my work has remained about the same”, or “Quality of my work has decreased.” This variable was dichotomized for analysis to (1) those who reported their quality of work has decreased and (2) those who reported their quality of work increased or remained the same. In addition, to garner more detailed information about their response, an open-ended follow-up question was asked “What is the reason for this change (or lack thereof)?” The item on the participant’s perceived enjoyment of work was presented in the same two-step process.

Additionally, the study’s research team developed four questions regarding the participant’s clients during the pandemic. First, providers were asked if they were conducting sessions with clients at the time of participation using the question “Do you conduct sessions (either group or one-on-one) with clients” (yes or no). Then, as three separate follow up questions, participants were asked, “How would you describe your client enrollment/attendance/attrition during this time compared to pre-COVID?” with the forced choice options of “No change”, “A little change”, or “A lot of change”. In order to further investigate the association of perceived change of work quality and enjoyment by type of work, participants were also asked “What is your approach to therapy?” and “What position do you currently hold at your job?”. Based on the responses of questions, we grouped participants into 3 mutually exclusive subgroups, including (1) evidence-based job (N = 38; LCSW, LCPC, Nurses) (2) non-evidence-based job (N = 34; support specialists (peer support specialists, housing support)), and (3) neither (N = 19; administration (CEO, assistant director)).

### Quantitative statistical analysis

We conducted bivariate analyses to test for differences in sample characteristics and impact of the COVID-19 pandemic on their work and clients (Table 1) between providers who reported their quality of work decreased during the COVID-19 pandemic relative to those who did not. Perceived change in quality of work was originally assessed as a 3-level categorical variable, including (1) decrease (N = 46); (2) about the same (N = 13); and (3) increase (N = 32). Given the small sample size we dichotomized this variable to improve the interpretability of results. Specifically, we collapsed “about the same” and “increase” into one group and “decrease” was the other group. T-tests were conducted for continuous variables and chi-square tests for categorical variables. If the cell count was less than 5, the Fisher’s exact test was performed. All analyses were conducted using SAS 9.4 [37]. Two-sided *P*-values of less than 0.05 were considered statistically significant.

**Table 1:** Sample characteristics and impact of the COVID-19 pandemic (*N* = 91)

	Total	Quality of work decrease (*n* = 46)	Quality of work same or increase (*n* = 45)	P-value
	*n* (%) or Mean (SD)			
Age (yrs.)				0.64
18–30	22 (24)	13 (28)	9 (20)	
31–50	49 (54)	23 (50)	26 (58)	
51+	20 (22)	10 (22)	10 (22)	
Gender				**0.01**
Cisgender Male	19 (21)	4 (9)	15 (33)	
Cisgender Female	71 (78)	41 (89)	30 (67)	
Other	1 (1)	1 (2)		
Race/Ethnicity				0.68
Caucasian	82 (90)	41 (89)	41 (91)	
African American	5 (6)	2 (4)	3 (7)	
Other	4 (4)	3 (7)	1 (2)	
Marital status				
Married	48 (53)	23 (50)	25 (56)	0.28
Other	43 (47)	23 (50)	20 (44)	
Education level				0.72
Lower than bachelor’s degree	21 (23)	9 (20)	12 (27)	
Bachelor’s degree	26 (29)	13 (28)	13 (25)	
Higher than bachelor’s degree	44 (48)	24 (52)	20 (44)	
Have any kids				
No	27 (30)	11 (24)	16 (37)	0.17
Yes	62 (70)	35 (76)	27 (63)	
Conduct sessions with clients				0.05
No	17 (19)	5 (11)	12 (27)	
Yes	74 (81)	41 (89)	33 (73)	
Client enrollment				**0.04**
A lot of change	34 (37)	22 (48)	12 (27)	
A little change/No change	57 (63)	24 (52)	33 (73)	
Client engagement				**0.04**
A lot of change	38 (42)	24 (52)	14 (31)	
A little change/No change	53 (58)	22 (48)	31 (69)	
Client attrition				**0.02**
A lot of change	24 (27)	17 (40)	7 (16)	
A little change/No change	66 (73)	29 (63)	37 (84)	
Enjoyment of work				
Increase	18 (20)	1 (2)	17 (38)	**< .001**
Same/decrease	72 (80)	44 (98)	28 (62)	
Workplace Stress (Mean, SD)	37.2 (18.0)	44.2 (18.9)	29.7 (13.6)	**< .001**
Experienced burnout (Mean, SD)				
Emotional Exhaustion (range: 0–36)	14.6 (7.6)	17.0 (7.7)	12.2 (6.9)	**0.002**
Personal Accomplishment (range: 0–32)	21.8 (3.4)	21.5 (3.3)	22.1 (3.5)	0.43
Depersonalization (range: 0–20)	3.0 (3.1)	3.2 (2.8)	2.7 (3.3)	0.37

### Qualitative analysis and mixed methods comparisons

Content analysis codes were derived using a combination of deductive and inductive approaches [3, 6, 41]. Using a deductive approach, we first generated initial codes derived from literature on health care provider burnout and experiences [5, 35, 42]. Next, two independent coders from the research team reviewed a subset of participant responses (n = 103) and an inductive approach was used to refine the initial codebook to align with additional themes identified in our dataset. Then, using the finalized codebook, each coder reviewed each participant response and coded it with one or more of the applicable themes. Because the COVID-19 pandemic and experiences of the providers were unprecedented and no literature existed to guide outreach and support for this group at the time this data was collected, we quantified the frequencies of each theme [13, 16] in order to highlight patterns in data (i.e., delineate those problems that were most commonly mentioned). For the purpose of this study, the thematic analysis framework was utilized. The end result of the thematic analysis was intended to highlight the most salient groups of meanings present within the qualitative data [32].

For the purpose of this study, during analysis, we were guided by the methodological triangulation design, specifically the transformation model [11]. As directed by the data transformation model, the quantitative and qualitative data were collected concurrently, and after the initial and independent analyses of these data, we subsequently transformed our qualitative data by quantifying the results (i.e., summed up the number of times that identical themes were mentioned) [11]. The quantitative and qualitative analyses occurred simultaneously with both of these methods being given equal weighting in their interpretation. Similar methods have been described in Creswell and Clark Plano (2007) which depict the transformation of open-ended qualitative questions on a survey as a valid triangulation mixed methods design whose purpose is to further support the quantitative findings by providing additional context based on rich participant qualitative responses [10, 17]. Because qualitative coding was completed without reference to quantitative survey responses to reduce bias, the third coder also reviewed any responses that were discrepant between the qualitative codes and the quantitative survey responses (n = 21; e.g., quantitative response indicated no change in work quality, but qualitative response indicated decrease in work quality). This decision to review responses was made as participants may have elaborated on their response or shared more nuanced and detailed perceptions regarding their experiences in the open-ended qualitative format versus the quantitative forced choice format, and we wanted our final dataset to be representative of all participant responses provided. Discrepancies between the quantitative and qualitative responses were minimal (n = 21 for quality of work, n = 10 for enjoyment of work) and were resolved by creating a composite variable taking each piece of data into account. Composite variables were created for both quality of work and enjoyment of work, and this quality of work variable was the final variable used for bivariate comparisons.

## Results

### Quantitative analysis results

Table 1 shows demographic characteristics of the 91 participants. The majority of this sample were women (78%), Caucasian (90%), and provided direct clinical care (81%). About half of participants were in the 31–50 age group (54%), married (53%), and obtained a graduate level degree (48%). Half of participants reported a perceived decrease in quality of work related to the COVID-19 pandemic. There were no significant differences in between demographic characteristics and self-perceived change in quality of work with one exception of gender (χ^2^ (1, N = 90) = 8.07, p = 0.01), excluded self-identified as others (N = 1).

The descriptive and bivariate analysis results exploring the impact of the COVID-19 pandemic are presented in Table 1. Specifically, compared with male providers, females were more likely to report having increased or remained the same in quality of work (OR: 5.13, 95% CI 1.55–17.00). Compared with providers who perceived their workload remaining about the same or increase, those providers who perceived workload decrease were associated with more emotional exhaustion (17.0(7.7) vs. 12.2(6.9) of same or increase; p = 0.002) and more of them reported no change or decrease on the enjoyment of work (97.8% vs. 62.2% of same or increase; χ^2^ (1, N = 90) = 17.78, p < 0.001). Providers who perceived decrease in quality of work were more associated with reporting a lot of change in client enrollment (48% vs 27% of same or increase, χ^2^ (1, N = 91) = 4.35, p = 0.04), engagement (52% vs 31% of same or increase, χ^2^ (1, N = 91) = 4.15, p = 0.04) and attrition (40% vs 16% of same or increase, χ^2^ (1, N = 91) = 5.09, p = 0.02), compared with providers who perceived same or increase in work quality. Perceived decreases in quality of work were not statistically associated with direct client work (as known as, conduct sessions with clients), although this association was approaching significance (89% vs. 73% of same or increase, χ^2^ (1, N = 91) = 3.74, p = 0.05). Additionally, significant differences were observed by type of work. Providers with neither non-evidence based nor evidence-based job were associated to report more about the quality of work remaining same or increase. And, providers with non-evidence based job were marginally significantly associated with higher workplace stress (p = 0.07), and emotional exhaustion burnout (p = 0.06) (as shown in in Additional file 1: Table S1).

### Qualitative analysis results

Qualitative analysis results supported our quantitative findings of high workplace stress and burnout rates among the providers queried (as shown in Table 2). The majority of responses indicated that perceived decreases in quality of work were due to increases in client needs or treatment barriers (19/31, 61%), feeling overworked with longer hours (5/31, 16%), and feeling emotionally exhausted or burned out (5/31, 16%). Similarly, less human contact and socialization (19/47, 40%), dealing with changes in work life balance and other personal issues (16/47, 34%), and leadership and management problems (10/47, 21%) were mentioned as causes for decreased enjoyment of work (Table 3). Challenging aspects of working during the COVID pandemic (Table 4) included both staff challenges they or other co-workers have experienced (38%) and challenges experienced when working directly to support clients (69%). Within staff specific challenges, both personal struggles during the pandemic that may have impacted work (16/33, 48%) and structural or logistics issues with remote work or longer hours (14/33, 42%) were noted. Challenging aspects of working during the pandemic tended to include lack of accountability [among staff] or contacting participants (27/60, 45%), difficulties establishing rapport via remote support options (14/60, 23%), and clients’ limited access to technology or limited digital literacy (12/60, 20%).

**Table 2:** Perceived quality of work during COVID-19

Open-ended responses (n = 72)		Participant quotes
Quality of Work Improved	17 (23%)^a^	"As unfortunate as COVID has been for our community, it allowed for creativity in our treatment with patients in both maintenance and enrollment. I provide OUD and Narcan training and I have been able to connect with many individuals/facilities through webinars."
Improved Organization	2 (12%)^b^	"Due to going mostly virtual, I have had to become much more reorganized to manage everyone that is also virtual."
More Worktime	3 (18%)^b^	"I have more time to plan/prepare for sessions and more time to spend with patients."
Fewer Distractions	3 (18%)^b^	"Staff can tend to personal needs as well as client's needs more directly, without the distractions and hold-ups of being on-site. Staff prefers to work alone."
Other	12 (71%)^b^	"Due to loosened telehealth and prescribing limitations."
Quality of Work Decreased	31 (42%)^a^	"[I] usually am motivated by working with the clients. Just talking to the clients over the phone does not provide for a feeling of accomplishment, on my part. Feel that I could help the client more in their progress through their recovery face to face as compared to through a phone that does not allow you to watch their body language and behaviors."
Work Overload/Longer Hours	5 (16%)^c^	"My workload has increased but productivity with clients has decreased because clients are not returning calls."
Client Needs & Barriers	19 (61%)^c^	"I have found it more difficult to challenge clients and broach difficult issues during phone sessions and telehealth sessions. I think this stems from my concern about client safety and a fear that they will terminate session and I have limited recourse to offer support/ensure safety from there. "
Stressed/Burned Out/Emotionally Drained	5 (16%)^c^	"I find telehealth and phone sessions more draining. I also feel like there's increased pressure to prove we are remaining productive during COVID."
Other	16 (52%)^c^	"Constant push to understand telehealth may be the new normal.”
No Difference in Work Quality	24 (32%)^a^	"No change."

^a^Percentage out of Total Excerpts (N = 72)

^b^Percentage out of Parent Code “Quality of Work Increased”

^c^Percentage out of Parent Code “Quality of Work Decreased”

Subcodes not mutually exclusive (n = 18 assigned more than 1 applicable code)

**Table 3:** Perceived enjoyment of work during COVID-19

Open-ended responses (n = 80)		Participant quotes
Enjoyment of work decreased	47 (59%)^a^	"Due to all the changes, the job has been made more difficult as we try to navigate circumstances that are out of our control."
Less Human Contact/Social Isolation	19 (40%)^b^	“It is less enjoyable because of lack of personal interaction with colleagues and patients."
Personal Issues	16 (34%)^b^	"Working from home has created a very difficult situation in which I am not able to define good boundaries between my work and personal life. Examples would be office phones and emails being forwarded to my personal phone, which could be at any hour."
Leadership/Management	10 (21%)^b^	"My company did not have a good plan in place and is not organized or prepared."
Other	17 (36%)^b^	"Lack of childcare, increased clinical load, decreased ability to work on my research (which is substance use related)."
Enjoyment of Work Increased	17 (22%)^a^	"It has made me appreciate my job even more than I did before. I love what I do and helping others and seeing the changes and the benefits daily is a wonderful feeling. I think COVID-19 has made people appreciate the smaller things in life more than they did before."
Commute Time	3 (18%)^c^	"Enjoyment has increased because of less stress due to commute and finding a parking place and saving on gas and car wear and tear."
Other	15 (88%)^c^	"Success in adapting care during very challenging times."
No difference in enjoyment of work	16 (20%)^a^	"I'm still essentially doing what I did while working before the pandemic, so my enjoyment hasn't changed."

^a^Percentage out of Total Excerpts (N = 80)

^b^Percentage out of Parent Code “Enjoyment of Work Decreased”

^c^Percentage out of Parent Code “Enjoyment of Work Increased”

Parent codes for enjoyment of work are not mutually exclusive (n = 10 assigned more than 1 applicable code); Subcodes not mutually exclusive (n = 17 assigned more than 1 applicable code)

**Table 4:** Most challenging aspects during COVID-19

Open-ended responses (n = 93)		Participant quotes
Staff Challenges	33 (38%)^a^	"Staff experiencing the same stress/community trauma alongside their clients. Every normal life stress has been 10 × harder."
Personal Challenges	16 (48%)^b^	"We're all struggling to stay motivated and positive."
Structural Challenges	14 (42%)^b^	"Performing several extra job duties that were never listed on employment application that are not being compensated for."
Other	5 (15%)^b^	"Managing fears of staff and patients in light of incompletely understood pandemic."
Client Challenges	60 (69%)^a^	“Connecting and engaging clients during phone sessions. Interpreting client message without the benefit of body language and eye contact.”
Lack of Accountability	27 (45%)^c^	"As great as telehealth has been for me as a clinician, the client can always ignore the phone call."
Difficulties Establishing Rapport	14 (23%)^c^	"The lack of personal interaction with patient while supporting his/her needs."
Technology Access/Digital Literacy Issues	12 (20%)^c^	"Getting them on the phone for their services due to lack of cell service, minutes, and poor or outdated phones."
Other	17 (28%)^c^	"Making sure that everyone is mindful of safety and educated on the safety guidelines."

^a^Percentage out of Total Excerpts (N = 93T)

^b^Percentage out of Parent Code “Staff Challenges”

^c^Percentage out of Parent Code “Client Challenges”

Parent codes for enjoyment of work are not mutually exclusive (n = 9 assigned more than 1 applicable code); Subcodes for challenges not mutually exclusive (n = 17 assigned more than 1 applicable code)

Some providers indicated that their work quality had increased during the pandemic; their primary reasons for this change included having more time to complete activities (2/17, 12%), fewer distractions (3/17, 18%), and improved organization (3/17, 18%). Similarly, fewer transitions and shorter (or absent) commutes were cited in some responses (3/47, 6%) for those who felt their enjoyment of work had increased.

## Discussion

The current COVID-19 pandemic has impacted people in the SUD treatment field drastically. Clinical and nonclinical staff are at risk of psychological distress as they were expected to adapt quickly to the new regulations and protocols while still providing the best quality of work for their clients. In the current study, many providers perceived that their work quality decreased during the COVID-19 pandemic, and this was often due to a lot of changes in client enrollment, engagement and attrition, compared with providers perceived that their work quality remained same or increase. Additionally, they reported significant decreases in work enjoyment, as well as greater workplace stress and emotional exhaustion. Qualitative methods provided specific insights about possible sources of stress, highlighting the increased risk and support needs of clients with SUDs during this time.

Nationwide literature demonstrates a sharp increase in demand for substance use treatment during the early stages of the pandemic, coupled with significant structural barriers to the delivery of these services, as well as an increase in relapse, overdose, and mental health risk among clients [18, 21, 27, 36]. These stressors at both the service delivery and client need levels may have contributed to the reported perceived decrease in work quality among providers surveyed in this study. These changes along with the rapid required transition to remote services, may have contributed to providers feeling as though they had less capacity to directly support their clients and less of an ability to impact their clients’ health outcomes and conditions due to virtual communication and distancing during the COVID-19 pandemic [9, 43]. Numerous staff reported therapeutic barriers to conducting virtual versus in-person appointments, difficulties making successful contact with clients, and challenges to keeping clients engaged and motivated in their treatment from a distance. Such obstacles can make it difficult for providers to remain at the forefront of their clients’ care and may lead to a subsequent decrease in their quality of work [24, 25]. Perceived decreases in work quality were significantly related to less enjoyment of work with many providers reporting a lack of personal contact, pressures to meet productivity with decreased client engagement, and increased responsibilities with minimal management support as factors. Thus, staff experiencing decreased quality in their work may also report an overall decrease in their enjoyment of work, which could have significant effects on not only the staff member and their clients, but also the organization as a whole. Addiction treatment facility leadership should work to ensure that additional support is readily available to employees during the pandemic including proper telehealth training, remote work materials, adequate personal protective equipment, and updated mental health and physical wellness resources to help alleviate burnout and foster feelings of teamwork and unity. SUD treatment providers may also benefit from continuous training, support, and mentorship in providing virtual care as telehealth care will likely remain an integral component of healthcare moving forward.

Furthermore, respondents with perceived decreased work quality also reported higher workplace stress and increased emotional exhaustion, a component of burnout. Emotional exhaustion and meeting clients’ needs were frequently reported as the most challenging aspect of the COVID-19 pandemic among our provider respondents, giving context for increased client needs or increased client risk during the pandemic due to limited available resources, social isolation, or technology issues or barriers that may be affecting the quality of their care [9, 43]. As there were no reported differences in services between respondents with lower versus higher or unchanged work quality, it may not have been that services decreased or clients could not access treatment, but that staff felt that the quality of these interactions and services were diminished during this time. Current and future burnout among addiction treatment providers may be mitigated by treatment facility leadership providing additional self-care resources, administration assessing and redistributing increased workloads, hours, and staff responsibilities, as well as providing interventions that focus on individual- and organizational-level strategies, mindfulness, stress management and small group discussion as these previously have proved successful for reducing employee burnout [15, 23, 44]. Considering the limitations on the benefits provided by individual self-care practices during the pandemic, systemic changes in the framework and compensation of services should also be made to ensure employees are guaranteed sufficient wages and benefits, proper working conditions, and adequate paid time off to help relieve some of the responsibility and burden felt by individual staff members.

While our study is one of the first to examine how the COVID-19 pandemic has impacted addiction treatment providers on their perceived enjoyment and quality of work, our findings should be considered in light of the following limitations: First, the timeframe of the study measured participants’ perception of the COVID-19 pandemic’s impact at various time points in the pandemic. As the pandemic and its impacts have been dynamic, participants in the beginning of data collection may not have had the same impact on those who participated at the end of the study. Second, we did not have a baseline for how participants felt about their quality of work, enjoyment of work, and client enrollment, engagement, and attrition prior to the pandemic. Other common factors that could have affected their quality and enjoyment of work, such as long-standing issues of low pay and high staff turnover, were not accounted for in this survey. Third, we created various COVID-19-related questions as there were no specific instruments published to measure the changes within their treatment facility which can lead to information bias. Fourth, we did not assess the participant’s personal life. We were unaware of how the COVID-19 pandemic and other psychological factors may have impacted their at-home life. Further, causation cannot be inferred from these findings. Our sample was not asked if the decreased perceived quality of work caused decreased enjoyment of work, or vice versa and were not asked to specify the directionality of enrollment changes at their facility (i.e., whether their caseloads increased or decreased). Finally, our sample is relatively small and only recruited participants from one state, therefore our results may not be generalizable to providers in more well-resourced, for-profit care settings, or to provider populations with a different demographic makeup than our predominantly Caucasian and female sample.

## Conclusion

Our study results indicate that workplace stress and burnout rates are high among SUD providers during the COVID-19 pandemic, due to both workplace and personal factors. These levels of stress not only impact providers themselves, but also their ability to provide quality care and support to clients they serve. As many individuals working in substance use care are also in recovery themselves, identifying increases in stress levels that may contribute to recurrence of substance misuse or other behavioral health concerns is crucial to mitigating these issues. As the COVID-19 pandemic slowly resolves in the coming months and years, SUD treatment administrators and oversight bodies should continue to gather information on the challenges faced by clinical staff in their respective programs and implement responsive changes to promote well-being among clinical providers and, subsequently, the people they aim to serve.

## Supplementary Information

**Additional file 1. Table S1.** Self-perceived change in quality of work by type of work (N = 91).

## Data Availability

The datasets used and/or analyzed during the current study are available from the corresponding author on reasonable request.
