# Supplementary material for: Impact of the COVID-19 pandemic on burnout and perceived workplace quality among addiction treatment providers
Source: Addict Sci Clin Pract. 2023 Jan 20;18:5. doi: 10.1186/s13722-023-00361-6 (PMC9854021; doi:10.1186/s13722-023-00361-6)
Supplement: Supplementary file 1 — Additional file 1. Table S1. Self-perceived change in quality of work by type of work (N = 91). [file 13722_2023_361_MOESM1_ESM.docx]

Table S1. Self-perceived change in quality of work by type of work (N = 91).

|  | Overall | Evidence-based Job  (N =38) | Non evidence-based Job  (N =34) | Neither  (N =19) | P |
| --- | --- | --- | --- | --- | --- |
| Self-perceived change of quality of work, N (%) |  |  |  |  |  |
| Quality of work decrease | 46 (51) | 19 (50.0) | 22 (64.7) | 5 (26.3) | **0.03** |
| Quality of work same or increase | 45 (49) | 19 (50.0) | 12 (35.3) | 14 (73.7) |  |
| Enjoyment of work, N (%) |  |  |  |  |  |
| Increase | 18 (20) | 8 (21.6) | 4 (11.8) | 6 (31.6) | 0.21 |
| Same/decrease | 72 (80) | 29 (78.4) | 30 (88.2) | 13 (68.4) |  |
| Workplace Stress (Mean, SD) | 37.2 (18.0) | 35.5(15.7) | 43.3(21.2) | 29.1(11.4) | 0.07 |
| Experienced burnout (Mean, SD) |  |  |  |  |  |
| Emotional Exhaustion (range: 0-36) | 14.6 (7.6) | 14.1(6.7) | 16.8(8.5) | 11.7(7.1) | 0.06 |
| Personal Accomplishment (range: 0-32) | 21.8 (3.4) | 22.5(2.9) | 21.5(3.8) | 20.8(3.3) | 0.41 |
| Depersonalization (range: 0-20) | 3.0 (3.1) | 2.9(2.8) | 3.4(3.5) | 2.2(2.7) | 0.39 |
